# Tracheal mast cell tumor in a dog – surgical approach and diagnosis

**DOI:** 10.29374/2527-2179.bjvm000525

**Published:** 2025-08-19

**Authors:** Fernanda Rezende Souza, Karen Yumi Ribeiro Nakagaki, Fernanda Freitas Miranda, Leonardo Dias Mamão, Geovanni Dantas Cassali

**Affiliations:** 1 Laboratório de Patologia Comparada (LPC), Instituto de Ciências Biológicas, Universidade Federal de Minas Gerais, Belo Horizonte, MG, Brazil; 2 Centro de Diagnóstico Veterinário – CELULAVET, Belo Horizonte, MG, Brazil; 3 Autonomous Veterinarian, Belo Horizonte, MG, Brazil

**Keywords:** dog disease, mast cells, neoplasia, ki67, c-KIT, doença de cão, mastócitos, neoplasia, ki67, c-KIT

## Abstract

Mast cell tumors (MCTs) are a type of cutaneous neoplasm prevalent in canines. Although less frequent, such neoplasms can involve other anatomical sites with no skin involvement, such as the trachea. The objective of the present case report is to describe the clinical, surgical, histopathological, and immunohistochemical features of a tracheal MCT in a dog. An 8-y-old, mixed-breed, male dog showed signs of dyspnea, coughing and choking. Tracheobronchoscopy revealed a mass in the cervical part of the trachea, almost completely obstructing its lumen. Surgery was performed for removal of the mass and part of the tracheal rings. Histologically, the trachea showed transmural thickening with a round cell neoplastic proliferation. Extracutaneous mast cell tumor was confirmed by toluidine blue staining. Immunohistochemistry was performed for c-KIT with KIT-staining II and Ki67 >23 cells/grid (and 73.2% positive cells). The dog exhibited no postoperative complications. A local recurrence occurred four months after surgery and the animal's general condition deteriorated, which led to the patient’s euthanasia. Although rare, mast cell tumors should be considered in the differential diagnosis of dogs with extracutaneous nodules and masses.

## Resumo

Os mastocitomas (MCTs) são um tipo de neoplasia cutânea predominante em caninos. Embora menos frequentes, essas neoplasias podem envolver outros locais anatômicos sem envolvimento da pele, como a traqueia. O objetivo deste relato de caso é descrever as características clínicas, cirúrgicas, histopatológicas e imuno-histoquímicas de um MCT traqueal em um cão. Um cão macho de 8 anos de idade, mestiço, apresentou sinais de dispneia, tosse e engasgo. A traqueobroncoscopia revelou uma massa na parte cervical da traqueia, obstruindo quase completamente seu lúmen. A cirurgia foi realizada para remover a massa e parte dos anéis traqueais. Histologicamente, a traqueia mostrou espessamento transmural com uma proliferação neoplásica de células redondas. O tumor de mastócitos extracutâneo foi confirmado pela coloração de azul de toluidina. A imuno-histoquímica foi realizada para c-KIT com coloração KIT II e Ki67 >23 células/grade (e 73,2% de células positivas). O cão não apresentou complicações pós-operatórias. Houve recorrência local e o estado geral do animal se deteriorou quatro meses após a cirurgia, sendo eutanasiado. Embora raros, os tumores de mastócitos devem ser considerados no diagnóstico diferencial de cães com nódulos e massas extracutâneos.

## Introduction

Mast cell tumors (MCTs) are of common occurrence in dogs and represent from 17.8% (Śmiech et al., 2018) to 21% of all the skin neoplasms in this species (Kiupel et al., 2011). Typically, this type of tumor is cutaneous (cMCT) or subcutaneous, with rare extracutaneous forms involving the gastrointestinal tract, oral mucosa, tongue, conjunctiva, salivary gland, nasal mucosa (Larsen et al., 2022), nasopharynx, larynx, spinal cord, urethra, liver, spleen, lung, (Iwata et al., 2000; Kiupel, 2017) and epididymis (Magalhães et al., 2023). To date, only two cases of tracheal MCT have been described in dogs (Carlisle et al., 1991; Harvey & Sykes, 1982). Extracutaneous MCTs (eMCT) appear to exhibit a more aggressive growth pattern than cMCT (Iwata et al., 2000).

A number of factors have been linked to the prognosis of MCT cases, including clinical staging, tumor location and size, intratumoral microvessel density, activating mutations of the c-KIT receptor, and markers of cell proliferation (Smith et al., 2017). The KIT expression patterns are available according to predominant staining, which can be classified as membrane-associated staining (KIT-staining I), focal or stipple cytoplasmic staining (KIT-staining II), and diffuse cytoplasmic staining (KIT-staining III) (Kiupel et al., 2004). KIT staining II and III are indicative of aberrant rKIT locations. These locations are associated with reduced survival rates and increased likelihood of recurrence (Kiupel et al., 2004; Romansik et al., 2007).

The cell proliferation markers used in MCTs are PCNA, Ki67, and AgNOR. In the case of Ki67, a number of different cutoff points have been established in the literature (Abadie et al., 1999; Scase et al., 2006). The use of multivariate analysis revealed that animals positive for Ki67 at >23 cells/grid area exhibited an elevated mortality rate associated with cMCT (Webster et al., 2007). The present paper presents a description of the clinical, surgical, histopathologic, and immunohistochemical features of tracheal MCT in a dog.

## Case description

**Figure 1:**
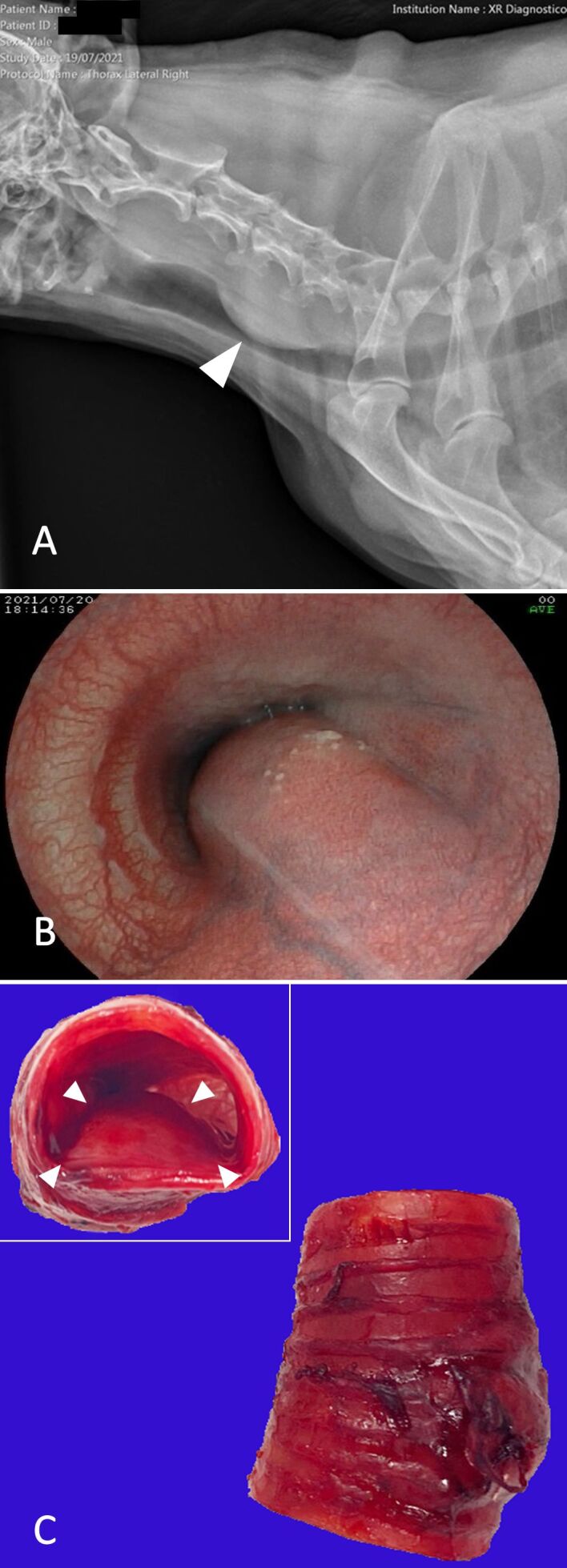
Canine, tracheal mast cell tumor. (A) Radiographic latero-lateral view of the cervical region reveals opacity area with increased intratracheal volume (arrowheads); (B) Tracheobronchoscopy with a mass in the cervical part of the trachea with a smooth surface causing almost complete obstruction of the lumen; (C) Tracheal section after complete removal of the mass. Inset: Section of the trachea showing the mass (arrowheads).

An 8-year-old, mixed-breed, male dog was referred to the veterinary clinic with progressive dyspnea, coughing, and choking. A latero-lateral radiograph revealed increased intratracheal radiopaque volume in the left cervical region, accompanied by a partial reduction in the tracheal lumen diameter (Figure 1A). Accordingly, the patient underwent tracheobronchoscopy examination. During the exam, a mass was identified in the cervical part of the trachea, measuring approximately 3.0 cm, leading to an intraluminal obstruction of 95%. The mass exhibited smooth surface, rigid texture, and sessile base (Figure 1B). No significant alterations were observed in the thoracic portion of the trachea, larynx, or bronchial region. Emergency surgery was conducted to excise the mass and nine adjacent tracheal rings (Figure 1C).

**Figure 2:**
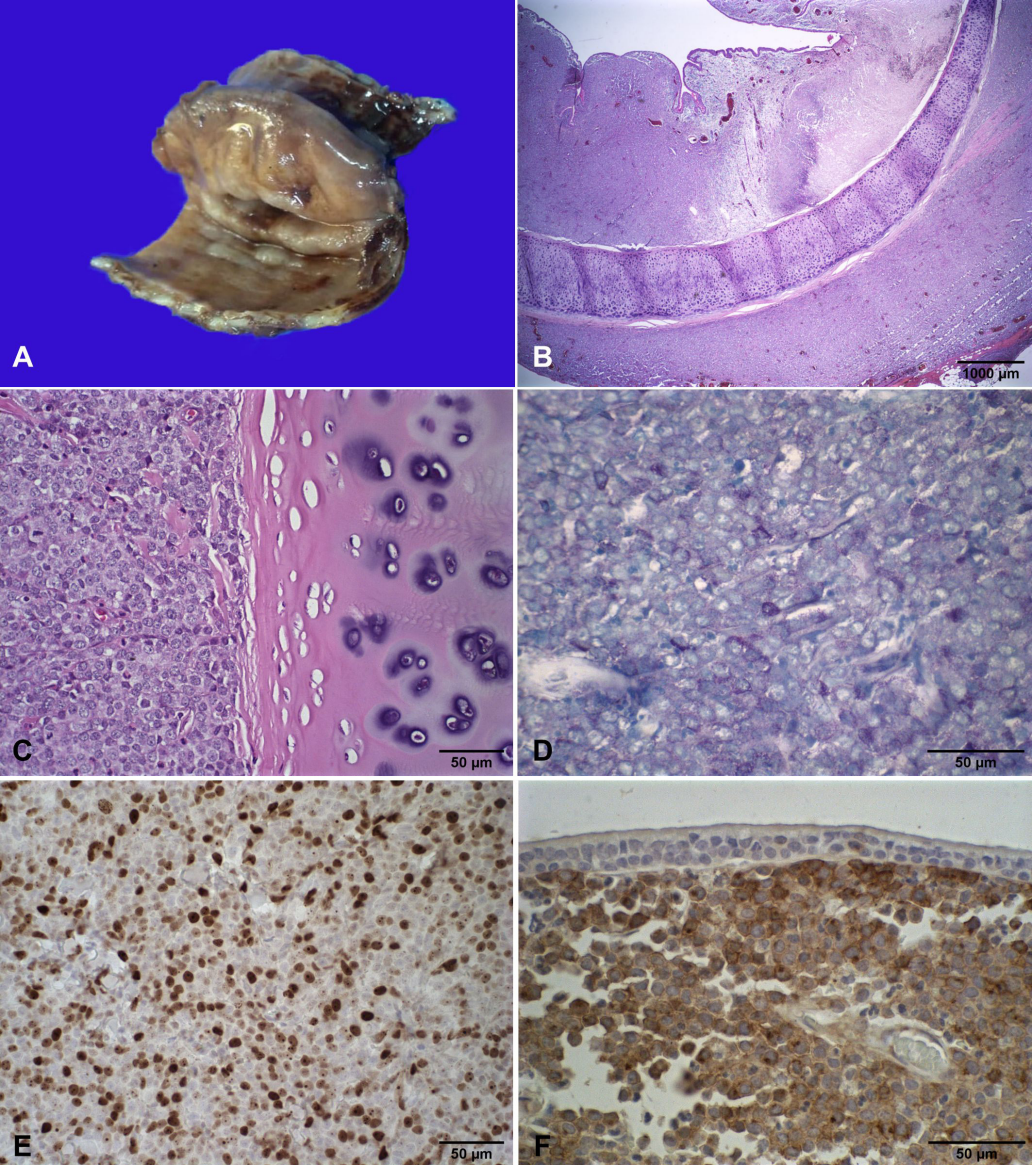
Canine, tracheal mast cell. (A) Irregular tracheal surface with a mass; (B) Round cell neoplasm transmural proliferation in the trachea (H.E., 2x objective, 1000 μm scale); (C) Proliferation of round cell neoplasm (left) and tracheal cartilage tissue (right) (H.E., 20x objective, 50 μm scale); (D) Metachromatic granules in the mast cell cytoplasm (Toluidine blue; 60x objective, 50 μm scale); (E) Neoplastic cells immunopositive for Ki-67 (IHC, 60x objective, 50 μm scale); (F) Neoplastic cells immunopositive for C-KIT with membrane-associated staining, diffuse cytoplasmic staining, and focal or stipple cytoplasmic staining (IHC, 60x objective, 50 μm scale).

The sample was fixed in formalin 10% and processed according to standard protocols. Macroscopically, a tissue fragment measuring 3.5 × 3.5 × 1.5 cm was observed, along with a mass of 2.3 × 2.0 × 0.3 cm. The fragment exhibited an irregular surface and firm consistency. The cut surface was solid, homogeneous, and brown (Figure 2A). Microscopically revealed notable transmural thickening with round cell neoplastic proliferation and sparse fibrovascular stroma (Figure 2B). The cells were round in shape, with moderately eosinophilic cytoplasm, moderately distinct cell borders, and a discrete to moderate number of granules. The nucleus was ovoid in shape, with finely granular chromatin and a distinct nucleolus. Notably, marked anisocytosis and anisocytosis were present, with occasional karyomegaly. Additionally, 22 mitotic figures in 2.37 mm^2^ (equivalent to 10 FN22/40X fields) were observed. Furthermore, a marked multifocal eosinophilic inflammatory infiltrate, multifocal areas of necrosis, hemorrhage, edema, and neovascularization were observed. The surgical margins were compromised (Figure 2C). Histochemical examination with toluidine blue was carried out to confirm the diagnosis and demonstrated the presence of metachromatic granules (Figure 2D).

Sections with a thickness of 4 µm were cut on gelatinized slides for immunohistochemistry (IHC). Antigen retrieval was performed in a water bath and under pressure heat according to a standardization already performed by the laboratory for canine samples. All reagents were used according to the manufacturer’s instructions. Endogenous peroxidase activity was blocked with 10% hydrogen peroxide (H_2_O_2_) for 15 minutes. Slides were incubated with monoclonal anti-Ki67 antibody (clone MIB-1; 1:50; DAKO) and polyclonal anti-CD117 or C-KIT antibody (1:800; DAKO) for 16 hours in a humidity chamber at 4°C. Incubation with secondary antibody and polymer was performed for 30 minutes each. Diaminobenzidine chromogen was used for 3 minutes and counterstained with Harris hematoxylin. Negative control was obtained by replacing the primary antibody with phosphate buffered saline (PBS). The evaluation of Ki67 and c-KIT was conducted by a similar classification system for cMCTs (Webster et al., 2006, 2007). The cell proliferative index (Ki67) was >23 cells/grid area and calculated by manual counting of 500 neoplastic cells, yielding a result of 73.2% (Figure 2E). The KIT expression pattern was identified as KIT-staining II with focal or stipple cytoplasmic staining in 30% of neoplastic cells (Figure 2F). No complications were observed following the surgical procedure. The patient survived for a period of four months postoperatively and was subsequently euthanized due to a deterioration in clinical condition and development of local recurrence.

## Discussion

Primary tracheal neoplasms are considered rare, and to the author's acknowledgment, this is the third case report of tracheal MCT in dogs. We use the literature on cMCT due to the lack of specific research on the eMCT and the similarity of histopathologic patterns. However, given the differences between cases, we recognize the limitations of this approach. There is no documented sexual or age predilection (Kiupel, 2017). In this case, it is a mixed-breed dog, although a higher predisposition is present in certain breeds, such as Boxer, Bull Terrier, French Bulldog, Golden Retriever, Labrador Retriever, Shar-Pei and Dachshund (Nardi et al., 2022).

In cases of tracheal tumors, the clinical signs may not be specific, and they may include stridor, vocal changes, exercise intolerance, cough, choking, dysphagia, weight loss, and anorexia. In the present case, the animal exhibited some of the signs, thereby underscoring the necessity for a detailed clinical examination and emphasizing the importance of imaging in the evaluation of respiratory signs. Tracheobronchoscopy examination was employed as auxiliary diagnosis. Sample collection for cytology brush is possible. Excisional biopsy is indicated in cases where the diagnosis cannot be made by cytology alone (Culp, 2020). However, in this case, the excisional biopsy was performed for histopathologic diagnosis, without prior sampling. Respiratory tract surgery is indicated in cases of neoplasm with the possibility of tracheal resection. Resection and anastomosis of eight to ten rings can be performed, depending on tracheal elasticity and tension (MacPhail & Fossum, 2021). In the present case, nine rings were surgically removed and no post-surgical complications were reported.

Despite the absence of a specific classification for eMCT, the 2-tier histologic grading system for canine cMCT (Kiupel et al., 2011) provides a framework for assessing this tracheal tumor. According to that system, the tumor would be classified as high-grade due to the presence of >7 mitotic figures in 2.37 mm^2^ (equivalent to 10 FN22/40X fields) and karyomegaly. High-grade cMCT was associated with lower survival time and reduced time to metastasis and new tumor development. Mitotic count (MC), obtained in hematoxylin and eosin staining, is a prognostic indicator in cMCT. Dogs with MC ≤5 had a longer survival time than dogs with MC >5, regardless of the grade of the neoplasia (Romansik et al., 2007). The same evaluation was performed in 33 dogs with oral mucosal MCT, which showed worse prognosis when MC >5 (Elliott et al., 2016). Similarly, the patient had an interval of approximately 120 days between diagnosis and local recurrence. However, due to the absence of an established grading system for eMCT and the limited number of cases of tracheal MCT, it is not possible to extrapolate the findings and ascertain the prognostic significance of MC in this neoplasm type.

In the present case, an eosinophilic inflammatory infiltrate, areas of necrosis, and edema were identified, consistent with the literature, which reports those common findings in cases of cMCT. Although not demonstrated in this case, collagenolysis, sclerosis, and secondary lymphocytic inflammation were also observed in cases of cMCT (Kiupel et al., 2011). In cases where there is scarce intracytoplasmatic granulation or the neoplastic cells are poorly differentiated, histochemical techniques are important tools to complete the diagnosis. Giemsa, toluidine blue, or alcian blue-safranin staining was performed to demonstrate metachromatic granules of neoplastic mast cells (Nardi et al., 2022). The utilization of toluidine blue staining served to confirm the diagnosis of MCT, while concurrently excluding the potential diagnoses of other round cell neoplasms, such as plasmacytoma, histiocytoma, or lymphoma.

A study of 39 canines diagnosed with gastrointestinal tract MCT revealed that immunohistochemical analysis yielded a positive result for c-KIT, with membrane-associated and cytoplasmic staining in 77% of cases (Nardi et al., 2022). In the present case report, we observed membrane-associated and cytoplasmic staining. The neoplasm showed KIT-staining II due to the predominant focal or stipple cytoplasmic staining, as indicated in the cMCT literature (Webster et al., 2006). The cell proliferation index, as determined by Ki67 evaluation, exceeded >23 cells/grid. Such finding has been associated with a worse prognosis in cMCT (Webster et al., 2007). In this particular case, the patient had a short time until recurrence. However, the scarce literature on eMCT does not allow us to extrapolate the information. The significance of KIT expression patterns and markers of cell proliferation in the prognosis of eMCT remains to be elucidated. Due to the rarity of eMCT, there is a paucity of knowledge regarding the behavior and treatment of this condition. Although rare, MCT must be included in the differential diagnosis of extracutaneous masses in dogs. The integration of imaging studies with histopathologic and immunohistochemical evaluation is paramount in establishing the diagnosis.

## Conclusion

In the present case, the association between clinical, anatomopathological, and immunohistochemical findings was essential to elucidate the diagnosis of eMCT. Although this type of neoplasm is considered rare in dogs, it should always be included in the differential diagnosis of respiratory masses. The present report will assist veterinarians in the diagnosis and management of such condition. Studies on eMCT are needed for a proper classification of this type of neoplasm.
